# A precision grading method for walnut leaf brown spot disease integrating hierarchical feature selection and dynamic multi-scale convolution

**DOI:** 10.3389/fpls.2025.1641677

**Published:** 2025-10-03

**Authors:** Yuting Wei, Debin Zeng, Liangfang Zheng

**Affiliations:** ^1^ College of Information Engineering, Tarim University, Alaer, China; ^2^ Key Laboratory of Tarim Oasis Agriculture, Ministry of Education, Tarim University, Alaer, China

**Keywords:** walnut, brown spot disease (*Ophiognomonia leptostyla*), hierarchical feature selection, edge features perception, adaptive multi-scale dilated convolution, disease grading

## Abstract

Walnut leaf brown spot disease, caused by *Ophiognomonia leptostyla*, is among the most destructive fungal diseases in walnut cultivation. In the development of smart agriculture, precision grading of plant diseases remains a core technical challenge; specifically, this disease is plagued by blurred lesion edges and inefficient extraction of complex features, which directly limits the accurate grading of the disease. To address these issues, this study proposes a disease grading method integrating hierarchical feature selection and adaptive multi-scale dilated convolution, and develops the CogFuse-MobileViT model. This model overcomes the limitations of the standard MobileViTv3 model in capturing blurred edges of tiny lesions via three innovative modules: specifically, the Hierarchical Feature Screening Module (HFSM) enables hierarchical screening of disease-related features; the Edge Feature Focus Module (ECFM) works in synergy with the HFSM to enhance the focus on lesion edge features; and the Adaptive Multi-Scale Dilated Convolution Fusion Module (AMSDIDCM) achieves dynamic multi-scale fusion of lesion textures and global structures. Experimental results demonstrate that the proposed model achieves an accuracy of 86.61% on the test set, representing an improvement of 7.8 percentage points compared with the original MobileViTv3 model and significantly outperforming other mainstream disease grading models. This study confirms that the CogFuse-MobileViT model can effectively resolve the issues of blurred edges and inefficient feature extraction in this disease, provides a reliable technical solution for its precision grading, and holds practical application value for the intelligent diagnosis of plant diseases in smart agriculture.

## 1 Introduction

Walnut diseases pose a serious threat to walnut production in Xinjiang. The spread of crop diseases significantly exacerbates the security risks of the walnut industry if timely prevention and control measures are not taken (Cooke, 2006; Khan et al., 2021). As a core component of the disease prevention and control system, early precise grading plays a critical role in agricultural production management (Adaskaveg et al., 2009; Chiang et al., 2016; Lamichhane, 2014). Walnut brown spot, caused primarily by the fungus *Ophiognomonia leptostyla*, typically forms specific symptoms on organs such as leaves, flowers, and fruits. Among them, leaves, as the primary carrier of plant diseases, exhibit characteristics such as lesion morphology and color changes on their surfaces, which often serve as important bases for disease grading (Ghaiwat and Arora, 2014; Weber, 1980; Zarei et al., 2019). Traditional disease identification relies on manual experience (Moragrega et al., 2011; Wang et al., 2020), a method that is not only time- color system to reduce interference from light and leaf veins, and lesion edge detection using the Sobel operator, ultimately achieving fast and accurate grading based on the ratio of lesion area to leaf area. Jadhav and Patil (2016) developed a leaf image partitioning technique based on k-means clustering and squared Euclidean distance, automatically quantifying damaged leaf area through the pixel ratio of lesions to leaves for disease grading. Arivazhagan et al. (2013) proposed a four-step processing system involving color conversion, green pixel removal, segmentation, and texture feature classification for leaf disease grading. However, traditional methods for acquiring disease information suffer from insufficient segmentation accuracy for plant leaf disease images with complex textures and unclear lesion boundaries, thereby resulting in poor disease severity grading performance.

With the continuous advancement of computational power, deep learning has increasingly become a key technology for addressing complex lesion segmentation, primarily due to its superior capability in automatic feature extraction (Mao et al., 2023). Chen et al. (2021) proposed the BLSNet method based on the UNet semantic segmentation network, enhancing lesion segmentation accuracy through the introduction of attention mechanisms and multi-scale feature fusion. Experiments showed that its segmentation and classification accuracy outperformed benchmark models such as DeepLabv3+ and UNet, preliminarily verifying the reliability of this method in automatic assessment of BLS disease severity. Ngugi et al. (2020) developed the KijaniNet segmentation network based on fully convolutional neural networks, demonstrating excellent performance in tomato leaf segmentation under complex backgrounds; Lin et al. (2019) developed a CNN semantic segmentation model that achieved high-precision pixel-level segmentation of cucumber powdery mildew on leaves. Additionally, some studies have fused traditional features with deep learning: Tripathi (2021) proposed a convolutional neural network method based on AlexNet, achieving disease grading by fusing features extracted by the model with external leaf segmentation features; Ma et al. (2017) introduced Comprehensive Color Features (CCF) combining hyper-red index, HSV/H and Lab/b components, and achieved lesion segmentation via interactive region growing, with experiments confirming that this method enables accurate grading of disease images such as cucumber downy mildew under actual field conditions. However, although such methods have improved grading accuracy to some extent, they still suffer from insufficient feature capture of blurred disease edges, making it difficult to achieve fine-grained differentiation of subtle differences between disease grades (Rastogi et al., 2015).

In recent years, the application of deep learning in plant disease classification has continued to expand. Parashar et al. (2024) systematically validated the capability of complex feature modeling for crop yield prediction, further substantiating the critical role of adaptive feature extraction in agricultural intelligent decision-making. Concurrently, Vishnoi et al. (2022) enhanced small-target detection precision in apple disease identification through a spatial attention mechanism-based CNN architecture for leaf disease diagnosis. Ozturk et al. (2025) constructed an ensemble learning classification model based on ResNet50, Mobile Net, EfficientNetB0, and DenseNet121, enhancing generalization performance through statistical cross-validation and improving decision interpretability via Grad-CAM visualization. Experimental results showed that the ensemble model achieved stable high-precision classification performance across multiple validation rounds. These studies provide robust and interpretable solutions for intelligent plant disease recognition through technical integration and architectural innovation. Hu et al. (2021) integrated Retinex enhancement, Faster R-CNN detection, and VGG16 classification to improve the grading and detection accuracy of blurred diseased leaves. Picon et al. (2019) proposed an adaptive deep residual neural network algorithm for the classification of three European wheat diseases (Septoria leaf blotch, brown spot, and rust) in real-world scenarios, which effectively improved the classification accuracy of wheat diseases. Kim and Ahn (2021) employed deep CNN architectures such as ResNet, Xception, and DenseNet, combined with transfer learning and fine-tuning, to classify 9 categories of pests, diseases, and healthy states in tomatoes. Although DenseNet combined with the RMSprop algorithm achieved an accuracy of 98.63%, single CNN architectures have clear bottlenecks in handling complex plant lesions and overlapping multiple diseases, including insufficient specificity in feature extraction and limited ability to distinguish subtle differences between lesions. Shi et al. (2023) analyzed the application of CNNs in evaluating the severity of plant diseases, pointing out that hybrid architectures such as classical CNNs, improved CNNs, and segmentation networks have limitations in handling complex plant lesions: classification errors caused by concurrent multiple diseases, as well as constraints on model generalization ability due to unbalanced datasets and insufficient annotation accuracy. These issues significantly restrict their precise application in practical agricultural scenarios.

Although significant advancements have been made in plant lesion segmentation and disease classification using deep learning, the grading task for walnut leaf brown spot disease—characterized by blurred edge representation and complex small lesions—still faces notable challenges. Current MobileViT-based hybrid models fall into two categories: general architectures (MobileViTv1/v2/v3) focus on optimizing the CNN-Transformer balance for natural images; domain-improved models (Mobile-Former/EdgeViT) are oriented toward medical/industrial tasks, with their task focus on localization rather than grading, which mismatches the pain points and fails to address the blurriness of agricultural lesions, tissue interference, and morphological variations. Thus, the present study proposes a novel CogFuse-MobileViT model specifically designed for disease grading, with its specific contributions as follows:

1. A diverse field-collected walnut leaf dataset was constructed, with images divided into four distinct severity levels based on qualitative assessment of disease severity, ensuring the accuracy of disease severity grading.
2. The Hierarchical Feature Selection Module (HFSM) enhances the fusion of local details (such as lesion texture and color) with global context through local and global attention mechanisms and task-driven feature selection, while suppressing interference from healthy regions.
3. The Edge Convolution Fusion Module (ECFM) strengthens edge details through Sobel convolution and residual connections, achieving effective integration of edge-specific features with general features, further enhancing edge details and enabling more precise capture of lesion contour details.
4. The Adaptive Multi-Scale Dilated Dense Inception Convolution Module (AMSDDICM) extracts differentiated features using multi-shaped convolution kernels and adaptively fuses multi-scale information via a dynamic weight mechanism, capturing lesion morphologies at different pathogenesis stages.

## 2 Materials and methods

### 2.1 Characteristics of walnut leaf brown spot and classification of disease severity levels

In the local standard Technical Regulations for Prevention and Control of Walnut Brown Spot (McGranahan and Leslie, 1991), walnut brown spot is divided into four severity levels. The severity of plant leaf diseases is generally determined using the spot coverage method. In this study, walnut leaves with different disease severities were processed to separate disease-infected spots from healthy leaf areas, and the percentage of lesion area to total leaf area was calculated. With reference to the local standard, the disease grades specified in the standard were re-determined through calculations in this study, as shown in **Table 1**. Classification of different severity levels of walnut leaf brown spot was conducted in accordance with Technical Regulations for Prevention and Control of Walnut Brown Spot (Wang et al., 2022; Yang et al., 2021).

**Table 1:** Walnut leaf brown spot disease severity grading criteria.

Disease grade	Symptom	Area ratio of S1 to S2 (K)
0	Absence of symptoms	0
1	Near-round or irregular-shaped small white spots	0<K<5%
2	Leaf disease spots become large, irregular black and brown	5%<K<30%
3	There are a large number of spots on the leaves, the spots fusion, forming a large area of coke	>30%

**Figure 1:**
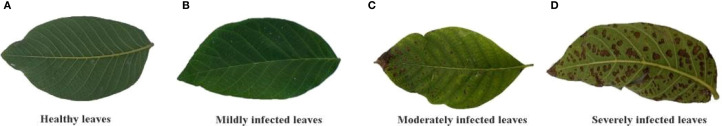
Walnut leaves infected with brown spot disease at different severity levels. **(A)** Healthy leaves; **(B)** Mildly infected leaves; **(C)** Moderately infected leaves; **(D)** Severely infected leaves.

Walnut leaf brown spot is caused by infection with the fungus *Ophiognomonia leptostyla* (Mircetich et al., 1980). In the early infection stage, near-circular or irregular small spots appear on the leaves, with a gray-brown center and dark yellow-green to purplish-brown edges, and diseased leaves tend to fall off prematurely. In the middle stage, elongated elliptical or irregular slightly sunken dark brown lesions form, with larger spot size and light brown edges; longitudinal cracks are often present in the center of the lesions. In the late stage, lesions often coalesce to form large scorched necrotic areas, surrounded by yellow to golden-yellow zones, and small black granules (conidiomata and conidia of the pathogen) are scattered on the surface of the diseased tissue (Mircetich et al., 1980). According to the degree of color and texture feature changes in infected leaves, walnut leaf disease is divided into four stages: healthy, early, middle, and late stages, corresponding to disease severity levels: healthy (Level 0), mild (Level 1), moderate (Level 2), and severe (Level 3). The different severity levels of walnut leaf brown spot are shown in **Figure 1**.

**Table 1** shows the ratio of walnut leaf brown spot lesion area to total leaf area. Level 0 (Healthy): Healthy leaves without symptoms. Level 1 (Mild): Near-circular or irregular small white spots appear on leaves, accounting for less than 5% of the leaf area. Level 2 (Moderate): Lesions expand into irregular dark brown spots, covering 5% to 30% of the leaf area. Level 3 (Severe): Abundant lesion coalesce to form large scorched necrotic areas, exceeding 30% of the leaf area.

### 2.2 Calculation algorithm for walnut leaf brown spot disease severity levels

**Figure 2:**
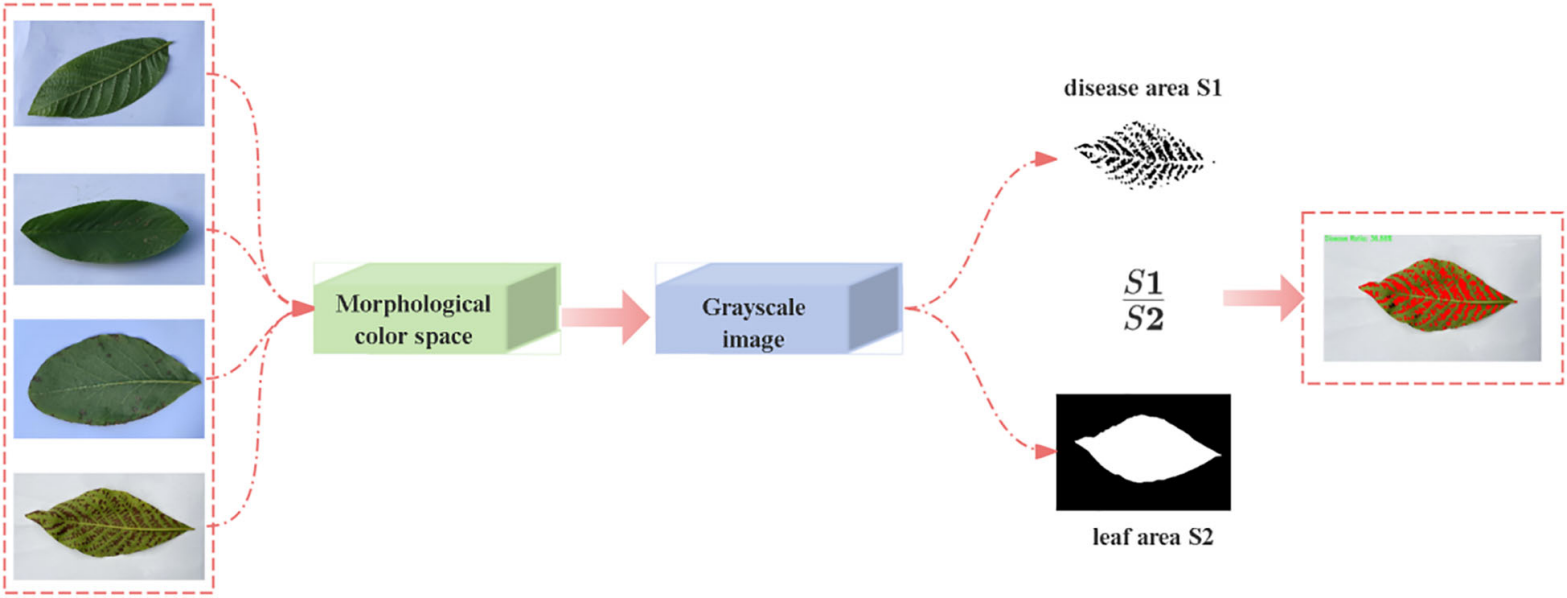
Computational method for different severity levels of brown spot infection in walnut leaves.

After capturing walnut leaf brown spot samples using a camera, to minimize subjective bias, this study strictly adhered to the objective quantitative criteria defined in the Technical Regulations for the Control of Walnut Brown Leaf Spot (**Table 1**), utilizing the lesion area percentage (K) as the primary grading indicator. we re-determined the severity levels through computational analysis, with the specific determination process shown in **Figure 2**.

First, a Python-based color segmentation algorithm was employed to convert images from the BGR color space to the HSV color space for processing. The HSV color space offers inherent advantages in color segmentation tasks. By analyzing the hue, saturation, and value characteristics of diseased regions, the color threshold range in the HSV space was determined (Chaudhary et al., 2012). After generating an initial disease region mask based on this threshold range, morphological operations such as closing and opening were applied to optimize mask quality, effectively eliminating noise while preserving the integrity of lesion contours. Subsequently, the original image was converted to grayscale mode, and the leaf region was extracted using an adaptive threshold binarization algorithm. Leaf boundaries were localized via contour detection technology, and a complete leaf mask was generated through contour filling. Pixel statistical analysis was performed on both the leaf mask and disease mask to calculate the total leaf area and diseased region area, respectively. When a valid leaf area (>0) was detected, the disease severity index was calculated using the following formula: Disease Severity Index $(\%)=\frac{S_{1}}{S_{2}}\times 100\%{\text{ S}}_{1}$ : Total Area of Diseased Regions ${\text{S}}_{2}$ : Total Area of the Complete Leaf.

### 2.3 Dataset construction

Data were collected from the Walnut Plantation Base of Tarim University (Alar, Xinjiang) between May and October 2024. Leaves from three locally dominant early-fruiting cultivars (‘Wen 185’, ‘Xinxin 2’, ‘Zha 343’)widely cultivated in southern Xinjiang were vertically photographed using an iPhone 13. This genotypic diversity ensures model generalizability by encompassing varied disease phenotypes. The orchard follows standardized cultivation with 4m plant spacing, 5m row spacing (≈50 plants/mu), and conventional management practices. Sampled trees were in full fruiting stage. To capture disease traits across microenvironments, samples were collected from safely accessible crown layers. The dataset includes healthy and brown spot-infected leaves three severity levels, spanning varied time periods, light conditions, and angles. After removing duplicates and invalid images,5,120 high-quality JPGs were retained for analysis.

#### 2.3.1 Development of the walnut leaf brown spot dataset

Disease severity grading was established through quantitative lesion area analysis (**Figure 2**). A representative subset of 512 images (10% of the full 5,120-image dataset) was selected via stratified random sampling, accurately preserving the original severity distribution. To ensure labeling rigor, two plant protection specialists (>5 years’ expertise in walnut pathology, certified in local protocols) executed standardized annotation: Pre-annotation training unified diagnostic criteria for ambiguous cases, with formal annotation commencing only after achieving pre-calibration inter-rater agreement (Kappa coefficient ≥0.75). As validated in **Table 2**, dual statistical metrics confirmed gold-standard reliability—inter-rater Cohen’s Kappa reached 0.71, meeting Landis & Koch’s “substantial agreement” threshold; algorithm-expert consensus attained a weighted Fleiss’ Kappa of 0.77, substantially outperforming conventional Cohen’s Kappa in multi-annotator scenarios. A three-stage standardization pipeline eliminated preprocessing discrepancies. The final dataset, partitioned into training/testing sets (8:2 ratio), strictly adheres to plant disease survey protocols and provides benchmarked data for deep learning model development (**Table 3**).

**Table 2:** Algorithm vs. expert consensus agreement evaluation.

Object of assessment	Number of samples (pieces)	Weighting scheme	Kappa coefficient	P
Algorithm vs Expert Consensus	512	Linear weighting a	0.77	<0.001
Inter-rater (Expert)	100		0.71	<0.001

**Table 3:** Walnut leaf brown spot disease image acquisition data.

Disease grade	Training set (amplitude)	Test set (amplitude)	Total number of samples (amplitude)	Label
0level	1167	292	1459	0
1level	1026	256	1282	1
2level	1031	258	1289	2
3level	945	236	1181	3
Total (amplitude)	4078	1042	5120	

### 2.4 Construction of walnut leaf brown spot disease severity grading model

#### 2.4.1 The optimized MobileViTv3 network: CogFuse-MobileViT

To address the challenge of difficult feature capture for small lesions in walnut leaf brown spot severity grading, this study proposes an innovative model, CogFuse-MobileViT. MobileViTv3 was selected as the backbone network due to its suitability for walnut brown spot grading. Its hybrid MobileNet-ViT architecture integrates CNN-based local feature extraction essential for lesion detail capture with Transformer-enabled global contextual modeling critical for analyzing lesion spatial distribution. This design aligns with pathological requirements for severity grading, necessitating concurrent attention to local lesion characteristics and global infection patterns. The lightweight architecture further supports real-time processing on embedded devices, enabling future field deployment.

**Figure 3:**
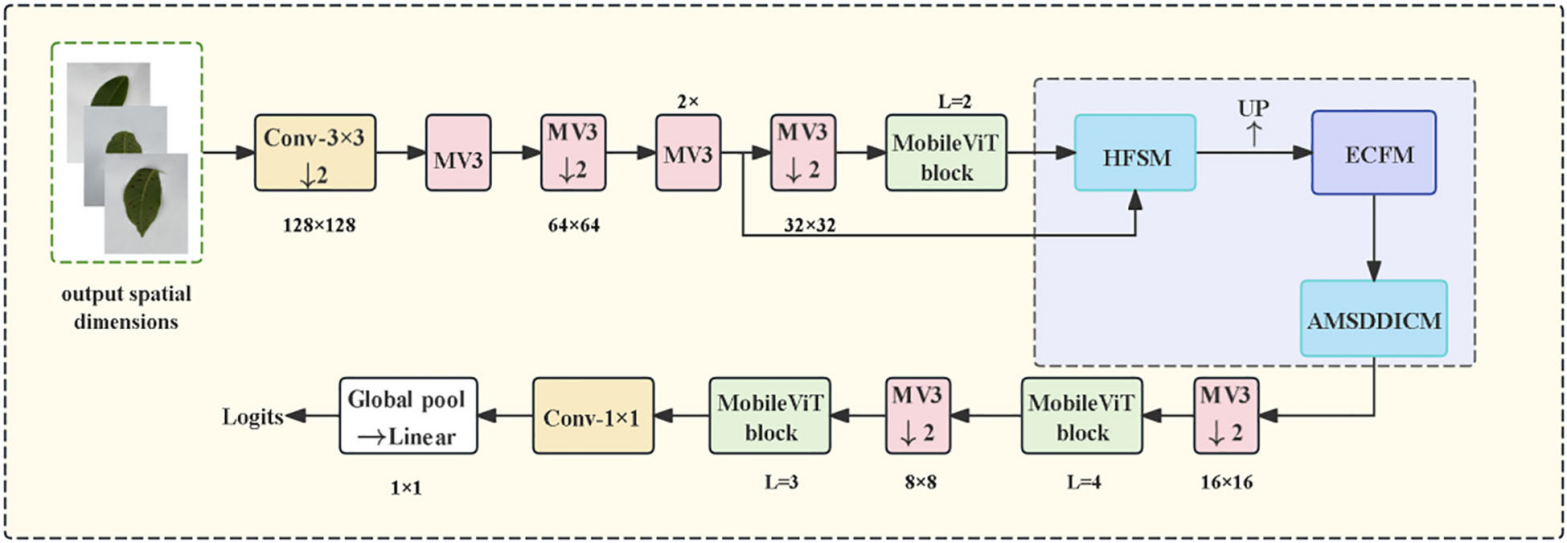
CogFuse-MobileViT Architecture Diagram (HFSM suppresses healthy regions through dynamic masks, ECFM explicitly extracts edges via Sobel convolution, and AMSDDICM fuses multi-scale features through dynamic weights).

The model embodies a “grading task-driven” design principle. Conceptually, it establishes a disease-specific framework of “hierarchical screening to edge enhancement to dynamic fusion.” This framework elevates general feature extraction to targeted solutions addressing three major agricultural challenges: Suppressing interference from healthy tissues via hierarchical screening resolving feature confusion; Strengthening blurred lesion contours through edge enhancement overcoming the bottleneck of edge blurriness; Adaptively handling multi-stage morphological variations using dynamic fusion addressing morphological variation challenges. Structurally, as illustrated in **Figure 3**, the network implements a progressive feature extraction strategy. The Hierarchical Feature Selection Module (HFSM) first fuses shallow and middle-layer features. It employs a hierarchical attention mechanism to enhance semantic consistency while preserving spatial details. The Edge Convolution Fusion Module (ECFM) then processes these features. It utilizes learnable Sobel operators to extract edge information, which is fused with conventional convolutional features via residual connections to augment edge perception capability. Finally, the Adaptive Multi-Scale Dilated Inception Convolution Module (AMSDDICM) enables adaptive processing of multi-scale edge features. This module is capable of both capturing fine edge changes in minute lesions and grasping the overall contour structure of large lesions, thereby comprehensively covering edge features across different developmental stages. Based on these enhanced edge features, the network accurately outputs the final disease severity grade.

#### 2.4.2 The Hierarchical Feature Selection Module

**Figure 4:**
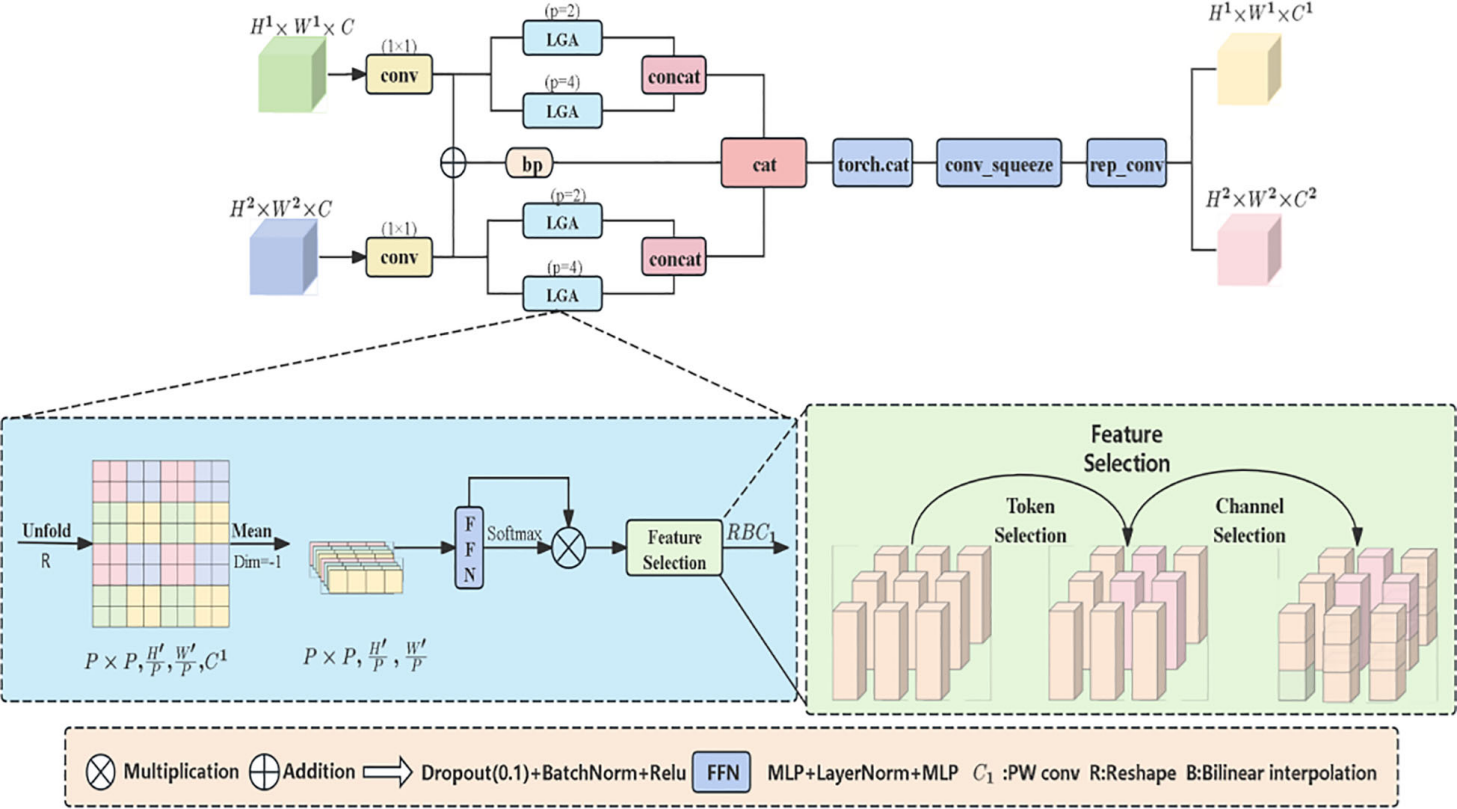
Architecture diagram of the hierarchical feature selection module (HFSM).


The HFSM (Hierarchical Feature Selection Module) is an innovative architecture proposed in this study. Unlike MobileViT’s direct feature concatenation, HFSM utilizes learnable prompt vectors (Equation 1) to generate spatial masks, dynamically suppressing non-lesion regions. Such task-driven selection is crucial for tiny objects. In the grading task of walnut leaf brown spot disease, this module utilizes a local attention mechanism to focus on micro-regions of walnut leaves, fusing shallow and middle-layer features. Meanwhile, the hierarchical feature selection mechanism enables precise capture of local detail features such as lesion texture and color, effectively suppressing interference from healthy leaf regions and enhancing disease features. This provides high-discriminative feature representations for brown spot disease grading while preparing for subsequent lesion edge processing. As depicted in **Figure 4**, the module processes two hierarchical feature maps. Initial 1×1 convolutions reduce both maps’ channels to half the output dimension curtailing computational load. One reduced map undergoes bilinear upsampling for spatial alignment with the other. These aligned features are summed and processed by a 3×3 convolution to generate base-path features. Simultaneously, the original reduced map and upsampled map are fed into dual Local-Global Attention modules for parallel processing (Xu, 2024). Each group contains local and global branches. During branch processing, the feature map is partitioned into P×P non-overlapping patches $P_{i,j}$ via the Unfold operation. After calculating the mean of pixel features within each patch, the result is processed using the following core formula: attention distribution generation.

(1) $${\text{Z}}_{\text{i},\text{j}}=\text{ML}{\text{P}}_{2}(\text{LayerNor}(\text{ML}{\text{P}}_{1}(\text{Mean}({\text{P}}_{\text{i},\text{j}}))))$$

(2) $${\text{a}}_{\text{i},\text{j}}=\text{Softmax}({\text{z}}_{\text{i},\text{j}})$$

Equations 1 and 2 convert the mean value of patch features into a high-dimensional feature vector through multi-layer perceptrons (MLP) and layer normalization (LayerNorm) (wherein( ${\text{MLP}}_{1}{\text{ and MLP}}_{2}$ ) ${\text{achieve dimensional transformation P}}^{2}\to \text{ouc}/2\to \text{ouc}/2$ )), and then generates the attention distribution via the Softmax function $a_{i,j}$ . This mechanism enables the model to focus on the key features of lesion regions, weaken the interference from healthy leaf areas, enhance the specificity of feature selection, and provide high-quality features for subsequent precise grading. After the local and global features output by the two modules are spliced along the channel dimension, they are combined with the basic path features to generate the output features of the final processing module.

For leaf images with over 80% healthy tissue, dynamic masks are generated via learnable prompt vectors to suppress green texture features while enhancing the gray-brown features of lesions (McGranahan and Leslie, 1991).

#### 2.4.3 ECFM Edge Convolutional Fusion Module

**Figure 5:**
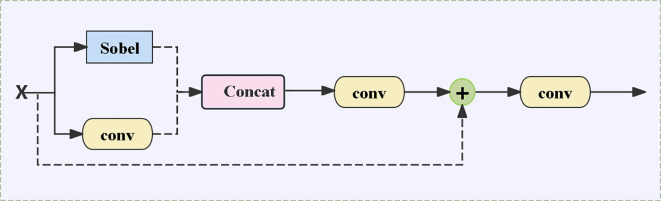
Architecture diagram of the edge convolutional fusion module (ECFM).


In the walnut leaf brown spot grading task, lesion edge features are crucial for accurately determining disease severity. The standard MobileViT relies on CNN-Transformer blocks to implicitly learn edges, whereas the ECFM (Edge Convolution Fusion Module) incorporates Sobel convolutions, conventional convolutions, and residual connections, effectively fusing edge features with general features and thereby enhancing edge details. As shown in **Figure 5**, the Sobel branch employs Sobel convolution to extract image edge information, accurately capturing the contour details of brown spot lesions; the convolutional branch captures general features such as leaf color and texture through standard convolution. The two realize feature fusion via the following core formula (Equation 3):

(3) S=SobelConv(x) 

Processing the input feature map (x) through the Sobel convolution operator (SobelConv) specifically extracts edge information of lesions (such as the boundary contours between lesions and healthy tissues, and the edge textures of small lesions). For the commonly seen blurred edges in brown spot disease, Sobel convolution can enhance edge gradient changes, making the originally blurred lesion contours clearer, thus providing key edge feature support for subsequent grading. Conventional feature extraction and fusion (Equation 4):


(4) $$\;\text{C}=\text{Conv}(\text{x}),\;{\text{F}}_{\text{concat}}=\text{Concat}(\text{S},\text{C})$$

 C=Conv(x) Extracting the overall features of leaves (such as the color distribution of lesions and the overall texture of leaves) through standard convolution forms a complement to edge features, preventing the model from focusing only on local edges while ignoring global lesion information.

${\text{F}}_{\text{concat}}=\text{Concat}(\text{S},\text{C})$ Concatenating the edge feature S and the conventional feature C along the channel dimension achieves the initial fusion of edge details and global features. This fusion enables the model to both identify the fine contours of lesions and judge the disease condition by combining the overall color and texture changes of lesions, thereby improving the accuracy of grading. The module also introduces feature addition and subsequent convolution operations: the fused features are first processed by the first convolution layer ${\text{F}}_{1}={\text{Conv}}_{1}({\text{F}}_{\text{concat}})$ , The corresponding operation results are added to the original input, and the final output is generated through the second convolution layer ${\text{F}}_{\text{final}}={\text{Conv}}_{2}({\text{F}}_{1}+\text{X})$ . In this process, the residual connection retains the original feature information, further strengthens the integration of edge features and conventional features, and ensures the effective transmission and enhancement of multi-level features.

The edge gradient information extracted by Sobel convolution (such as grayscale differences between diseased spots and healthy tissues) and the texture features from conventional convolution (such as the roughness of necrotic areas) are fused via residual connections. This not only preserves the blurred edges of early-stage lesions but also prevents edge features from being disconnected from the overall texture (Ghaiwat and Arora, 2014).

#### 2.4.4 AMSDDICM Adaptive Multi-scale Dilated Depthwise Inseparable Convolution Module

**Figure 6:**
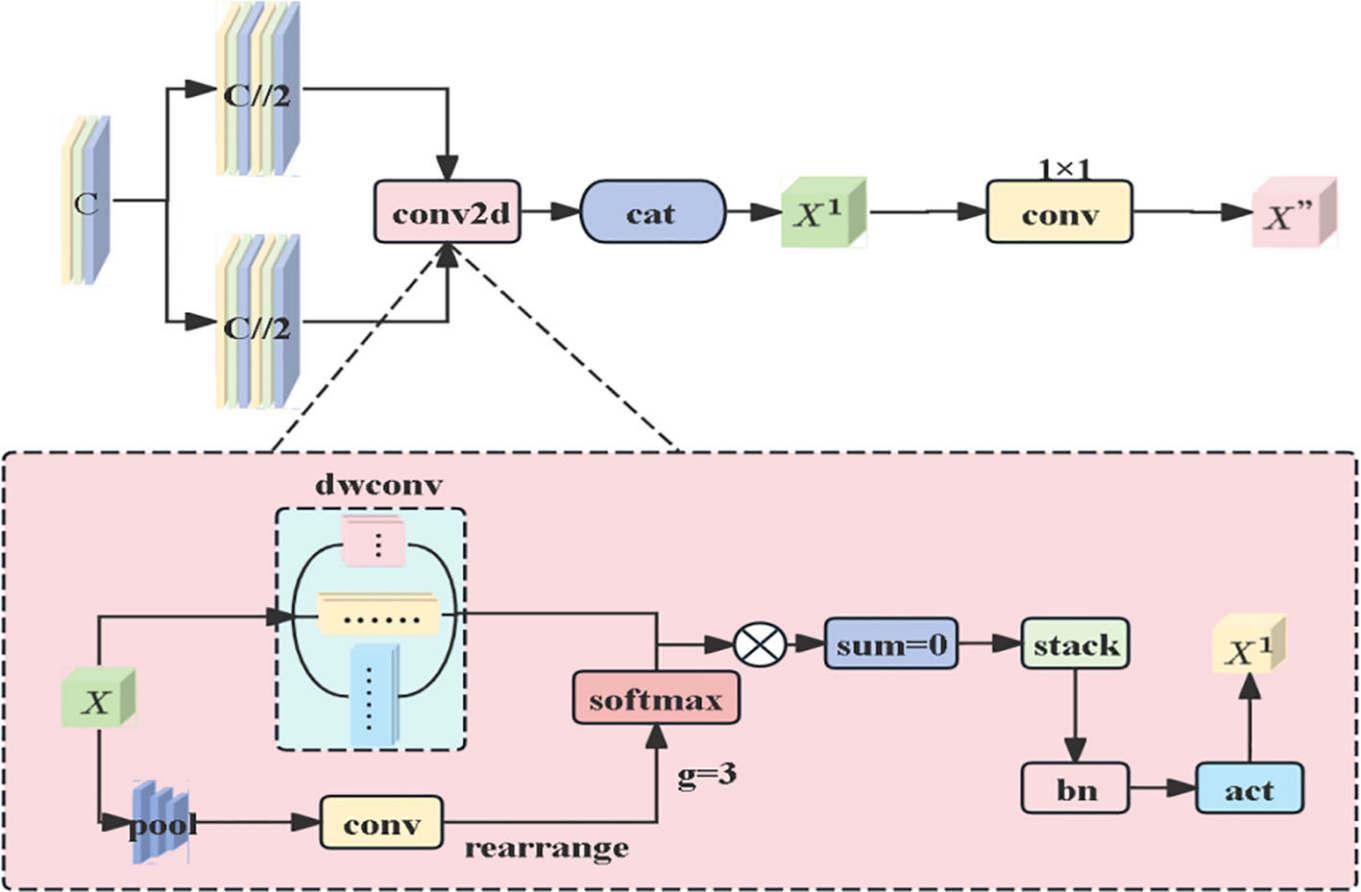
Architecture Diagram of the Adaptive Multi-Scale Dilated Depth wise Inseparable Convolution Module (AMSDDICM).

This module differs from MobileViT’s single-scale convolution, leverages multi-shaped convolution kernels to accurately extract lesion features of circular, irregular, and other shapes from multiple dimensions, enabling the identification of subtle differences in lesion edges and internal colors to enrich feature dimensions. Meanwhile, depth wise separable convolutions are employed to accommodate lesion scales at different stages of disease development. Specifically, the input features are first split into two groups, each entering the AMSDDICM module to extract features via multi-scale depth wise separable convolutions. A dynamic weighting mechanism (global pooling + convolution + softmax) is used to generate weights for fusing multi-scale features. The processed features from the two groups are concatenated, and finally, cross-channel fusion is completed via 1×1 convolution to output the final optimized features. The entire process integrates multi-scale convolution, dynamic weight allocation, and feature fusion to enhance the model’s ability to capture complex features, as shown in **Figure 6**. In response to the morphological differences between early-stage small spots (1-3mm in diameter) and late-stage fused spots (over 20mm in diameter), the dynamic weight mechanism enables the model to adaptively allocate the contributions of 3×3 kernels for capturing local textures and 11×1 kernels for extracting strip-shaped spreading features. This addresses the limitation of fixed weights in traditional Inception architectures in adapting to multi-scale morphologies.

When the input feature tensor X with the number of channels c enters the AMSDDICM module, it first undergoes a feature grouping operation: the input features are evenly split into two groups along the channel dimension, with each group containing half of the original number of channels (c//2). This grouping strategy not only reduces computational complexity but also creates conditions for subsequent multi-scale feature extraction. Each feature group is fed into an independent DMSconv2d module, which adopts different configurations such as 3×3, 5×5 square convolution kernels and 1×11, 11×1 strip-shaped convolution kernels — the square kernels capture local textures, while the strip-shaped kernels extract direction-sensitive long-range dependencies. This dynamic weight allocation draws inspiration from adaptive strategies in agricultural IoT systems. similar to how salinity-aware ETs models prioritize soil conductivity features under salt stress (Zhang et al., 2023), our mechanism selectively amplifies lesion morphological features critical for severity grading. The dynamic weighting mechanism of DMSconv2d generates weights through global average pooling and 1×1 convolution, with the core formulas as follows:

Weighted basic feature generation:


(5) $${\text{x}}_{\text{dkw}}=\text{rearrange}\left({\text{Conv}}_{1\times 1}\left(\text{AvgPoo}l2\text{d}\left(\text{x}\right)\right)\right)$$

Equation 5 compresses the spatial dimensions of the input features through global average pooling (AvgPool2d), retains global statistical information (such as the overall distribution characteristics of lesions at different scales), and then transforms the dimensions via 1×1 convolution to generate base features for calculating dynamic weights. This step provides a basis for subsequent weight allocation, enabling the model to preliminarily evaluate the importance of features at different scales. Weight normalization:


(6) $${\text{x}}_{\text{dkw}}=\text{F}.\text{softmax}({\text{x}}_{\text{dkw}})$$

Equation 6 compresses the spatial dimensions of the input features through global average pooling (AvgPool2d), retains global statistical information, such as the overall distribution characteristics of lesions at different scales, and then transforms the dimensions via 1×1 convolution to generate base features for calculating dynamic weights. This step provides a basis for subsequent weight allocation, enabling the model to preliminarily evaluate the importance of features at different scales. Weight normalization:


(7) $$\text{x}=\sum_{\text{i}=0}^{2}(\text{dwcon}{\text{v}}_{i}(\text{x})\times {\text{x}}_{\text{dkw},\text{i}})$$

Equation 7 is based on dynamic weights ${\text{x}}_{\text{dkw},\text{i}}$ ) for different convolution kernels ${\text{ dwconv}}_{\text{i}}(\text{x})$ Weighted fusion is performed on the extracted features. For walnut brown spot lesions exhibiting size heterogeneity and morphological complexity—ranging from early-stage circular micro-lesions to late-stage coalesced irregular lesions—this mechanism adaptively modulates multi-scale feature contributions. It prioritizes retention of morphology-discriminative features, local textures in small lesions or directional distributions in large lesions, thereby comprehensively capturing pathological characteristics across developmental stages. The processed features undergo channel-wise concatenation to generate optimized outputs (Mircetich et al., 1980).

### 2.5 Experimental process for severity grading of walnut leaf brown spot disease

**Figure 7:**
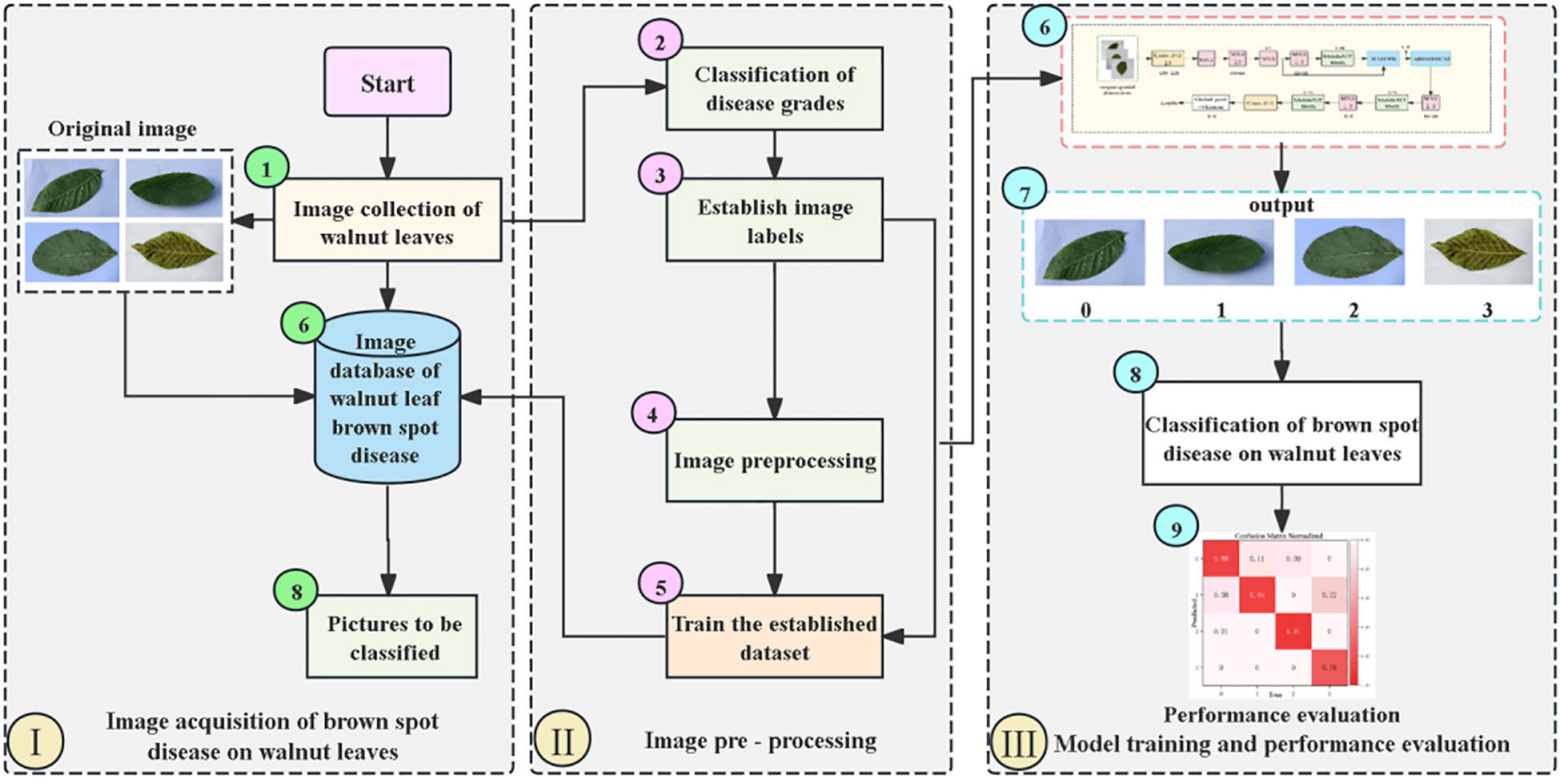
Overall Flow Chart for Severity Grading of Walnut Leaf Brown Spot Disease.

**Figure 7** is the overall flow chart of severity grading of walnut leaf spot disease. First is the image acquisition stage, where raw images of walnut leaves are captured and collected to construct an image database containing pictures to be classified (Singh et al., 2019). Next is the image preprocessing stage, which involves sequentially calculating and classifying disease severity levels, establishing image labels, and performing image preprocessing operations. Subsequently, the processed data are used to train the constructed dataset. Finally, in the model training and performance evaluation stage, the preprocessed images are input into the model for classifying walnut leaf brown spot disease, After which performance evaluation is conducted on the model’s classification results to determine the model’s effectiveness and accuracy.

### 2.6 Experimental parameters and evaluation metrics

#### 2.6.1 Test environment and hyperparameter setting

The experimental setup of this study is based on deep learning technology and leverages high-performance computing resources. All experiments were conducted on the Windows 10 operating system. The hardware platform consists of an AMD Ryzen 7 3700X processor and an NVIDIA RTX 2080Ti graphics card. The software environment was built using Python 3.8, the PyTorch 1.13.0 deep learning framework, and the CUDA 11.3 parallel computing platform. Additionally, PyTorch 1.13.0—a widely adopted open-source deep learning library renowned for its high flexibility—was selected, making it well-suited for research and development.

During model training, multiple adjustments were made to the hyperparameters to compare test results and select the optimal hyperparameter combination. The model accepts input images sized at 224×224 pixels, with a configured batch size of 32 during training and a maximum of 100 training epochs. The optimizer employs an initial learning rate of 0.003.

#### 2.6.2 Evaluation metrics

This paper aims to evaluate the performance of the model and verify the effectiveness of the improvement measures. We selected multiple evaluation metrics, and all subsequent experimental results adopt the method of calculating averages via 5-fold cross-validation, aiming to comprehensively and reliably evaluate the model performance. including precision (P), recall (R), etc. (Equations 8–16) These metrics can be calculated using the following formulas.

The arithmetic mean of the metric values across the five folds:


(8) $$\bar{\text{m}}=\frac{1}{5}\sum_{\text{i}=1}^{5}{\text{m}}_{i}$$

(9) Precision=TP/(TP+FP)

(10) Recall=TP/(TP+FN)

(11) $$\text{F}1\text{score}=\frac{2\text{TP}}{2\text{TP}+\text{FP}+\text{FN}}$$

(12) $$\text{Macro Precision=}\frac{1}{\text{C}}\sum_{\text{i}=1}^{\text{C}}{\text{P}}_{i}$$

(13) $$\text{Macro Recall=}\frac{1}{\text{C}}\sum_{\text{i}=1}^{\text{C}}{\text{R}}_{i}$$

(14) $$\text{Weighted Avg Precision=}\frac{\sum ({\text{N}}_{i}\cdot \text{Precisio}{\text{n}}_{i})}{\sum {\text{N}}_{i}}$$

(15) $$\text{Weighted Avg recall=}\frac{\sum ({\text{N}}_{i}\cdot \text{Precisio}{\text{n}}_{i})}{\sum {\text{N}}_{i}}$$

(16) $$\text{Weighted Avg Recall=}\frac{\sum_{\text{i}=1}^{\text{C}}\text{T}{\text{P}}_{i}}{\text{N}}$$

## 3 Experiments and results analysis

### 3.1 Core module design and validity experimental verification

#### 3.1.1 Comparative test of necessity of HFSM module

To validate the necessity of the Hierarchical Feature Selection Module (HFSM) within the CogFuse-MobileViT framework, this study conducted comparative tests against two mainstream lightweight attention modules: SE (Squeeze-and-Excitation) and CBAM (Convolutional Block Attention Module). As illustrated in **Table 4**, the HFSM module achieves a significantly higher accuracy of 86.61%, outperforming the SE module and CBAM module by 7.38% and 4.46%, respectively. This demonstrates that its hierarchical feature selection mechanism more effectively captures discriminative features. In terms of computational efficiency, the parameters and FLOPs of HFSM remain comparable with those of the SE module and CBAM module, indicating that its performance breakthrough stems from innovative structural design under equivalent lightweight constraints. Comprehensive results confirm that replacing HFSM with SE or CBAM modules would incur a performance degradation exceeding 4%, thereby validating the indispensable value of HFSM in enabling efficient feature selection within the CogFuse-MobileViT framework.

**Table 4:** Performance comparison of attention modules within the CogFuse-MobileViT framework.

Method Type	Accuracy (%)	Params (M)	FLOPs(G)
SE Module	79.23	2.00	2.09
CBAM Module	82.15	1.94	1.99
HFSM Module	**86.61**	2.02	2.10

Measured on NVIDIA RTX 2080Ti GPU (PyTorch 1.13.0, CUDA 11.3) with 224×224 input.

The bold font is used to highlight the performance of the HFSM and illustrate the necessity of the module.

#### 3.1.2 Convolutional kernel selection in AMSDDICM

Rigorous validation via kernel combination ablation studies (**Table 5**) demonstrates. The hybrid configuration (3×3 **+** 5×5 **+** 1×11) significantly outperformed square-only kernels (3×3 **+** 5×5) Accuracy 86.61% and 82.15% Stage-specific recall gains. Mid-stage lesions (Level 2) raise 9.4 percentage points. Late-stage lesions (Level 3) raise 6.1 percentage points. This empirically validates the necessity of 1×11 rectangular kernels for capturing linear pathological features, overcoming the limitation of isotropic kernels in detecting anisotropic structures.

**Table 5:** Comparison of different convolution kernels in AMSDDICM.

Method Type	Accuracy (%)	Level2 Recall (%)	Level3 Recall (%)
3×3	79.32	68.5	62.1
3×3 + 5×5	82.15	74.3	67.5
3×3 + 5×5 + 1×11	**86.61**	**83.7**	**73.6**

Level 2/3 corresponds to intermediate/late stage lesions.

The bold font is used to highlight the performance advantages of selecting specific convolution kernels.

#### 3.1.3 The impact of new modules on computational complexity

**Figure 8:**
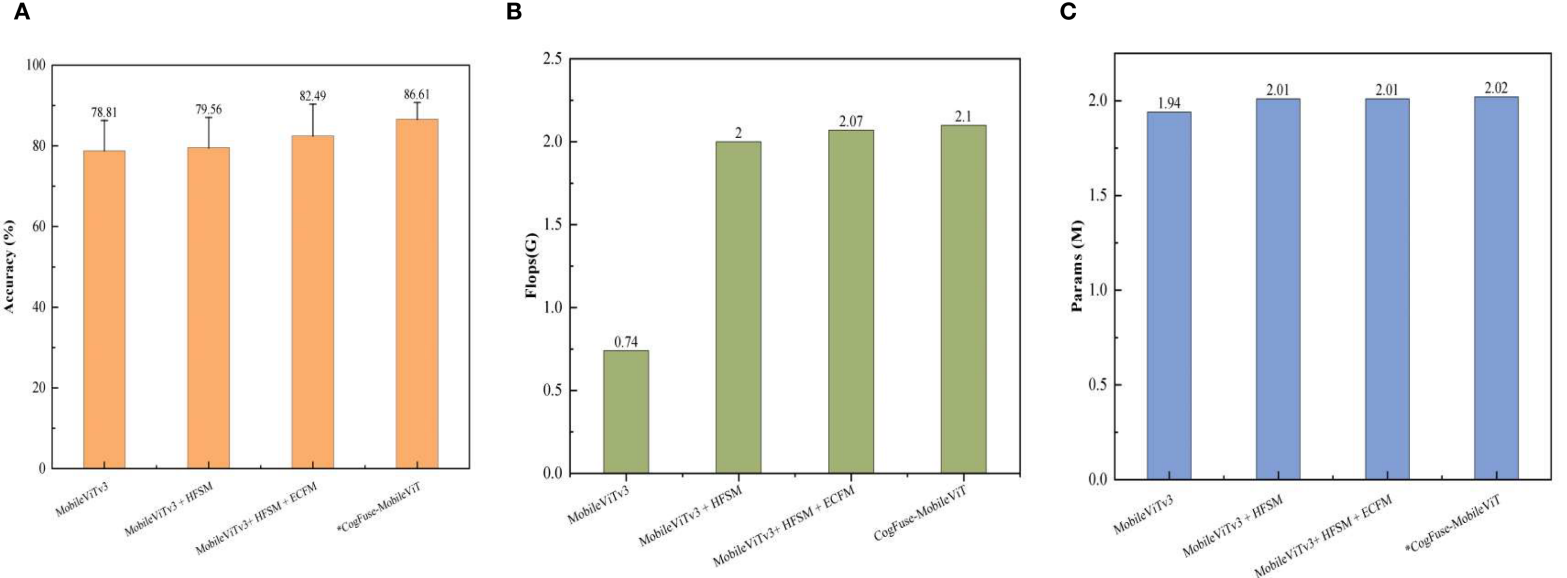
Comparison of the impact of each newly added module on computational complexity **(A)** accuracy (%); **(B)** Flops (G); **(C)** Params (M). Measured on NVIDIA RTX 2080Ti GPU (PyTorch 1.13.0, CUDA 11.3) with 224×224 input.

**Figure 8** shows that the HFSM module incurs a 169% FLOPs increase to achieve a breakthrough improvement in early-stage lesion detection—reducing Level 0/1 misclassification by 24%, thereby establishing the pathological foundation for severity grading. The ECFM module contributes a mere 3% FLOPs increment yet drives a 3.68 percentage point accuracy gain through enhanced edge feature representation. With only a 0.028G FLOPs overhead 1.3%, the AMSDDICM module enables adaptive fusion of multiscale pathological deformations, culminating in a 7.80-pp accuracy leap. These modules form a cascaded optimization paradigm: HFSM’s substantial cost resolves the core pathological bottleneck, while subsequent modules deliver superlinear returns—harvesting 6.85-pp accuracy gain with just 14% additional FLOPs—collectively establishing the globally optimal computation-performance equilibrium.

#### 3.1.4 Comparison of the influence of different module fusion on model performance

To validate the effect of module fusion, **Table 6** compares model performances with different combinations. When only HFSM (Hierarchical Feature Selection Module) is introduced, precision increases from the baseline of 82.23% to 83.77%, but recall decreases by 3 percentage points to 72.31%, causing the F1 score to slightly decline to 78.04%. Accuracy rises to 82.15%, indicating improved overall classification correctness of the model, but with potential risk of missed detections.

**Table 6:** Impact of fusion of different modules on model performance.

Model	Module	Level 0 F1(%)	Level 1 F1(%)	Level 2 F1(%)	Level3 F1(%)	Overall F1(%)	Precision (%)	Recall (%)	Accuracy(%)
Model 1		83.10	76.56	75.78	80.37	78.95	82.23	75.26	78.81
Model 2	HFSM	85.60	73.20	76.50	78.10	78.04	83.77	72.31	79.56
Model 3	HFSM+ECFM	87.80	79.80	80.60	81.20	80.51	86.34	74.69	82.49
Model 4	HFSM+AMSDDICM	86.30	80.50	81.30	82.10	80.79	85.34	76.24	83.94
Model 5	ECFM+AMSDDICM	84.10	78.80	79.70	80.80	81.82	89.38	74.27	81.33
CogFuse-MobileViT	**Tri-module collaboration**	**93.99**	**82.57**	**84.28**	**85.54**	**86.60**	**92.24**	**80.99**	**86.61**

The bold font is used to highlight the impact of the collaborative fusion of the three modules on the model performance.

When HFSM and ECFM (Edge-Context Feature Module) are synergistically introduced, precision increases to 86.34%, recall recovers to 74.69%, and both F1 score and accuracy are significantly optimized. HFSM fuses shallow and middle-layer features through a hierarchical attention mechanism, laying the foundation for subsequent edge feature processing; ECFM enhances lesion edge features. The combination of the two effectively improves the integrity of feature representation and the clarity of lesion boundaries.

The combination of HFSM and AMSDDICM (Adaptive Multi-Scale Dilated Depthwise Inseparable Convolution Module) further pushes recall to 76.24%, accuracy to 83.94%, and F1 score to 80.79%, outperforming the HFSM+ECFM combination. AMSDDICM compensates for HFSM’s deficiency in local feature refinement through attention-guided multi-scale detail fusion, which integrates multi-layer detail feature weights, especially suitable for scenarios with small targets or blurred features.

When the three modules work synergistically, all indicators reach optimal levels: precision, recall, F1 score, and accuracy increase by 12.19%, 7.67%, 9.62%, and 10.00% respectively compared with the baseline. Among them, HFSM lays the foundation for cross-layer feature fusion, ECFM effectively integrates edge-specific features with general features to enhance image edge information, and AMSDDICM adaptively fuses multi-scale and multi-type features through an attention mechanism, forming a progressive optimization chain from “hierarchical feature extraction” to “edge semantic enhancement” and then to “multi-layer detail fusion”. The experimental results show a balanced improvement in both precision and recall, indicating that the fusion of the three modules enables the model to focus more on learning the features of small brown spot lesions, thereby improving its classification performance. This synergistic task-specific design, combining hierarchical selection, explicit edge enhancement, and adaptive multi-scale fusion, fundamentally distinguishes CogFuse-MobileViT from prior MobileViT hybrids designed for general vision or localization tasks.

#### 3.1.5 Influence of different module combinations on F1-score of level (0-3)

In order to verify the targeted improvement of each module for a specific disease level, we conducted the following comparative experiments as shown in the **Table 6**, When HFSM is introduced alone, the F1-score of Level 0 increases from 83.10% to 85.60%, effectively reducing feature confusion between healthy leaves and early-stage lesions. After adding ECFM, the F1-score of Level 1 rises from 73.20% (with HFSM alone) to 79.80%, significantly mitigating the recognition bias caused by blurred edges of small lesions. AMSDDICM increases the F1-score of Level 3 from 80.37% to 85.54%, enhancing the adaptability to the morphology of large-scale fused scorched areas. With the collaboration of the three modules, the F1-scores of Level 0–3 reach 93.99%, 82.57%, 84.28%, and 85.54% respectively, achieving balanced optimization of performance across all levels.

### 3.2 Results comparison of different algorithms and statistical significance verification

#### 3.2.1 Comparison of grading results for different classification models

To verify the effectiveness of the CogFuse-MobileViT model, we selected 9 commonly used classification models to compare with the optimized CogFuse-MobileViT model, and the results are the average of 5-fold cross-validation over 120 epochs. As shown in **Table 7**, the proposed CogFuse-MobileViT model achieved the highest grading performance in terms of Precision, Recall, and F1-score for identifying walnut leaf brown spot disease at different severity levels among all compared models. The improved CogFuse-MobileViT model exhibits an accuracy of 86.61%, representing a 7.80-percentage-point improvement over the original model. Overall, the experimental results highlight that the enhanced CogFuse-MobileViT model is more conducive to focusing on lesion edge details and accurately learning the features of different severity levels of walnut leaf brown spot disease, thereby improving the model’s classification performance.

**Table 7:** Comparison of grading results for different classification models.

Models	Disease grade	Precision/(%)	Recall/(%)	F1/(%)	Accuracy/(%)
DenseNet	0	77.40	67.60	72.40	–
	1	64.70	75.90	74.50	–
	2	62.60	68.30	71.60	–
	3	81.30	53.31	55.15	–
	all	71.50	66.27	68.41	68.72
EfficienNet	0	71.40	71.37	66.44	–
	1	64.80	78.75	79.06	–
	2	56.50	53.16	63.29	–
	3	74.32	80.97	78.85	–
	all	66.75	71.06	71.91	69.91
EfficienNetV2	0	63.69	60.82	56.41	–
	1	78.05	70.00	76.95	–
	2	82.57	74.47	61.30	–
	3	80.30	68.09	63.88	–
	all	76.15	68.35	64.63	69.71
MobileNet	0	78.33	80.52	79.63	–
	1	71.14	59.12	65.93	–
	2	64.31	71.53	79.64	–
	3	84.91	60.81	71.67	–
	all	74.67	68.00	74.21	72.30
ResNet	0	61.54	71.23	66.03	–
	1	65.00	70.78	77.02	–
	2	61.33	75.38	75.32	–
	3	86.05	63.51	63.51	–
	all	68.48	70.22	70.47	69.72
ResNeXt	0	78.60	86.30	69.81	–
	1	66.79	69.84	63.04	–
	2	51.76	68.90	70.17	–
	3	64.44	64.27	72.49	–
	all	65.40	72.33	68.88	68.87
RegNet	0	73.38	65.97	76.25	–
	1	72.71	43.79	72.16	–
	2	70.34	62.00	65.85	–
	3	97.66	71.39	84.21	–
	all	78.52	60.78	74.61	71.30
Swin Transformer	0	67.50	67.40	83.10	–
	1	89.48	78.28	72.56	–
	2	77.65	72.66	40.78	–
	3	89.07	67.52	77.87	–
	all	80.93	71.47	68.53	73.63
MobileViTv3	0	87.30	78.40	83.10	–
	1	77.91	75.64	76.56	–
	2	72.65	75.54	75.78	–
	3	81.07	71.82	80.37	–
	all	82.23	75.26	78.95	78.81
CogFuse-MobileViT	**0**	**96.14**	**86.21**	**93.99**	**-**
	**1**	**92.98**	**80.47**	**82.57**	**-**
	**2**	**88.71**	**83.70**	**84.28**	**-**
	**3**	**91.13**	**73.60**	**85.54**	**-**
	**all**	**92.24**	**80.99**	**86.60**	**86.61**

The bold font is used to emphasize the advantages of the improved model in various metrics.

#### 3.2.2 Comparison of performance and reliability validation of different algorithms

**Figure 9:**
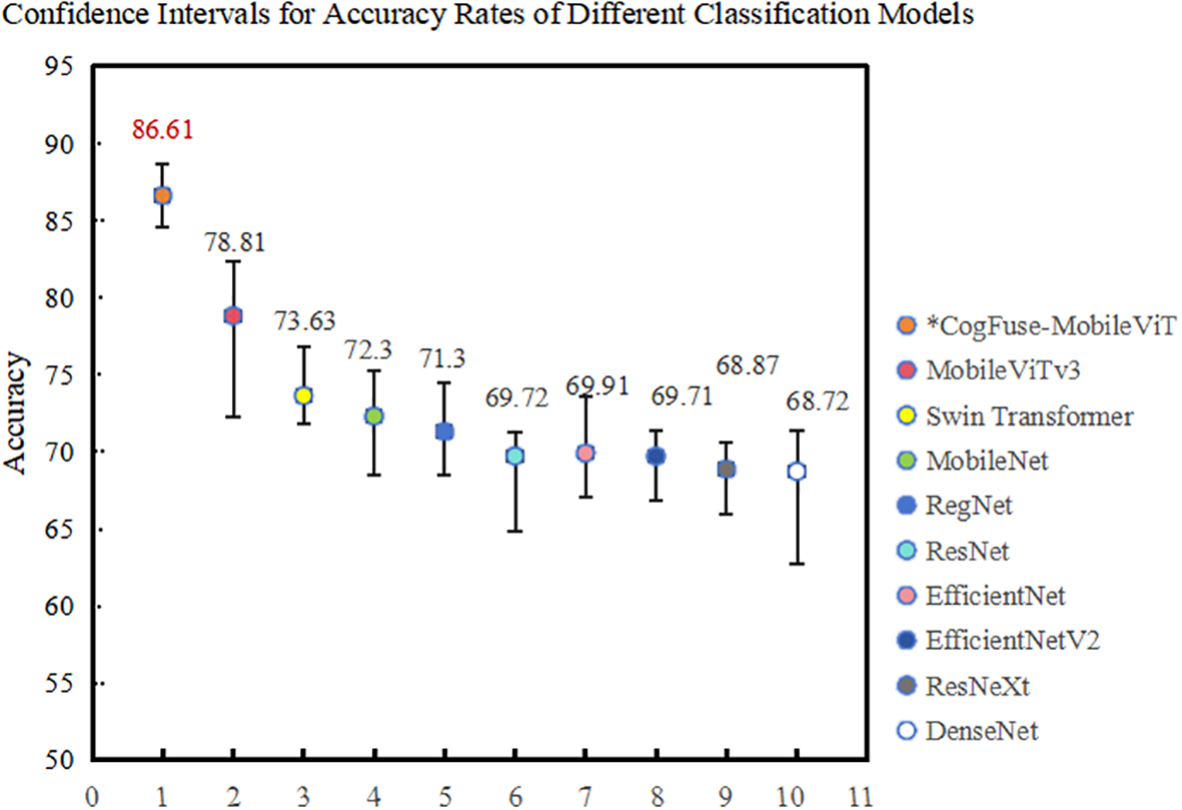
Confidence intervals of accuracy for different classification models in 5-fold cross-validation.

In model training, to quantify the reliability of results and avoid random biases from single experiments, this study employs 5-fold cross-validation to generate 95% confidence intervals, as shown in the **Figure 9**. The results demonstrate that CogFuse-MobileViT takes a significant lead with an accuracy of 86.61% (95% CI: [85.24%, 87.89%]). Its narrowest confidence interval range indicates the strongest generalization capability. Among the comparative models, MobileViTv3 has a 95% CI of [77.20%, 80.42%]; its lower bound is significantly lower than that of CogFuse-MobileViT, confirming that the 7.8% performance improvement is not due to random fluctuations. Furthermore, the confidence intervals constructed through 5-fold cross-validation further highlight the reliability of the experimental results.

#### 3.2.3 Statistical significance verification of model improvement

To statistically evaluate the performance improvement of CogFuse-MobileViT over the baseline MobileViTv3, an independent samples t-test was conducted (**Table 8**). For each model architecture, five independent training runs. The null hypothesis stated that the mean accuracy of CogFuse-MobileViT was equal to that of MobileViTv3 $H_{0}:\mu_{C\text{og}}=\mu_{Mob}$ ), while the alternative hypothesis stated that CogFuse-MobileViT had a higher mean accuracy ( $H_{1}:\mu_{C\text{og}}>\mu_{Mob}$ )CogFuse-MobileViT achieved a mean accuracy, significantly outperforming MobileViTv3. The independent samples t-test confirmed this improvement (t(8)=18.92,p=3.7×10 ^−8^).

**Table 8:** Comparison of accuracy results between MobileViTv3 and CogFuse-MobileViT using independent samples t-test.

Model	Average accuracy	SD	t(8)	P
MobileViTv3	78.81%	± 0.32%	–	–
CogFuse-MobileViT	**86.61%**	**± 0.28%**	**18.92**	**3.7×10^−8^**

Because of heteroskedasticity (Levene test: F=6.34, p=0.03), Welch t-test was adopted.

The bold font is used to emphasize the effect of statistical significance testing on the improved model.

**Figure 10:**
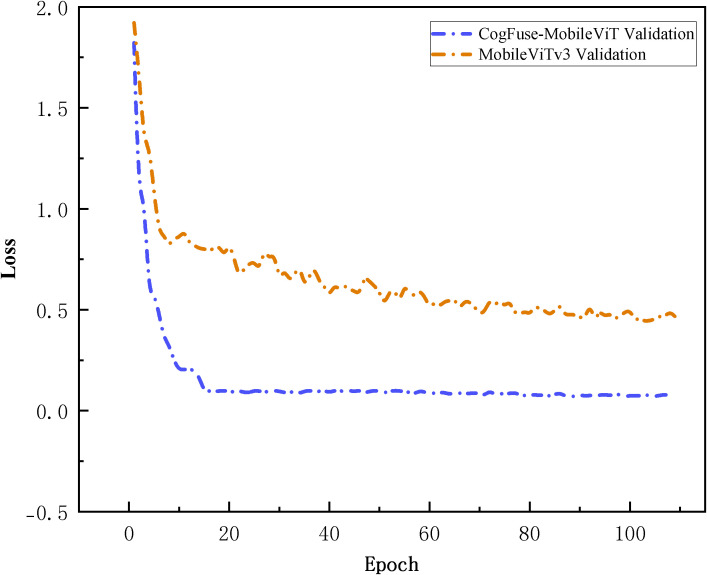
Comparison of loss curves between the CogFuse-MobileViT model and the original model in 5-fold cross-validation within 120 epochs.

Under 5-fold cross-validation (**Figure 10**), CogFuse-MobileViT achieves rapid convergence within the first 20 epochs, demonstrating more efficient feature learning capability. Upon entering the steady-state phase, its validation loss is reduced by over 5-fold compared to the baseline MobileViTv3, directly confirming smaller prediction errors and superior generalization capability. Meanwhile, the smooth and minimally fluctuating loss curve of CogFuse-MobileViT reflects strong robustness against data noise and distribution variations. Combined with the previous statistical findings from accuracy confidence intervals and independent t-tests, these results collectively validate the statistical significance of the improved model.

### 3.3 Model result analysis performance comparison of different models

**Figure 11:**
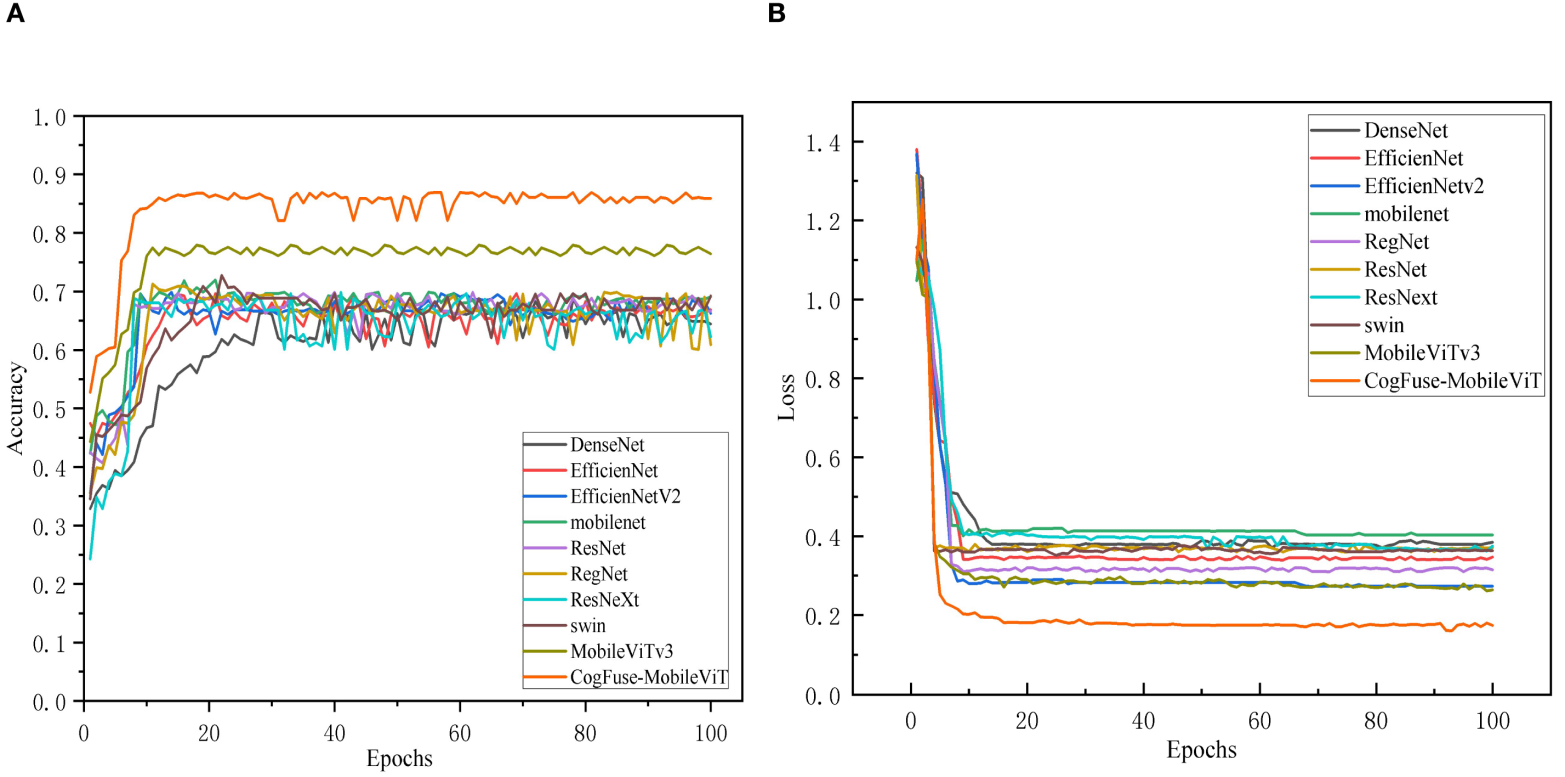
Comparison of Accuracy and loss across different Models **(A)** accuracy Line chart; **(B)** loss line chart.

**Figure 11A** shows that in the walnut leaf brown spot disease severity grading task, the classification accuracy of all models gradually improved and stabilized with the increase of training epochs, indicating that the models continuously optimized during learning until convergence. CogFuse-MobileViT stood out prominently: it achieved rapid accuracy improvement, reached a high level in relatively early training epochs with minimal subsequent fluctuations, and stably maintained the highest accuracy, demonstrating fast convergence and strong generalization ability to efficiently extract features distinguishing different disease levels. DenseNet also maintained high accuracy in the late training stage, but its accuracy improvement was slower, with slightly inferior convergence speed and final stability compared to CogFuse-MobileViT. Models like ResNet and RegNet showed limited accuracy gains with gentle upward trends, and their final stable accuracy values were significantly lower, reflecting insufficient feature extraction capabilities possibly due to network architecture or parameter optimization efficiency.

In **Figure 11B**, all models showed high initial loss values that dropped rapidly and then stabilized, following the typical learning process of iterative optimization. CogFuse-MobileViT achieved significant early loss decline and stabilized near the minimum, indicating efficient feature learning and strong fitting capability. While DenseNet also reached a relatively low loss level, it remained slightly higher than CogFuse-MobileViT. Other models had higher late-stage loss values: some showed fast initial decline but converged to higher levels, indicating shortcomings in capturing key disease features.

### 3.4 Analysis of detection results for different classification models

**Figure 12:**
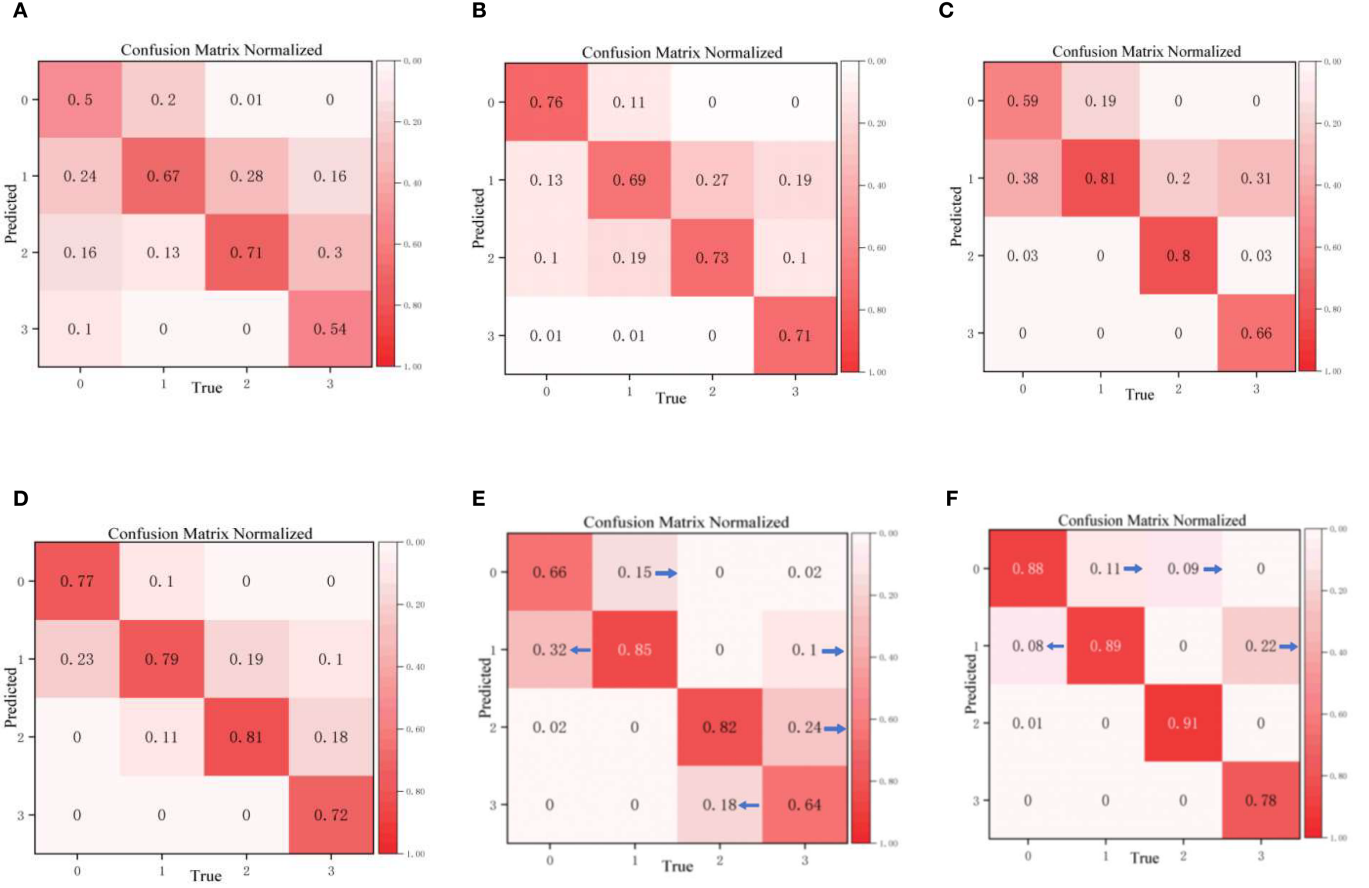
Confusion matrices of the improved cogfuse-mobilevit model and traditional classification models **(A)** DenseNet; **(B)** EfficientNet; **(C)** EfficientNetV2; **(D)** Swin Transformer; **(E)** MobileViTv3; **(F)** CogFuse-MobileViT.

The confusion matrix is a key indicator for evaluating classification models: the higher the diagonal values, the higher the classification accuracy for the corresponding category, and the lower the off-diagonal values, the fewer misjudgments. **Figure 12** shows the classification results of different models for the four disease grades (0–3) of walnut leaf brown spot disease. It is clearly evident that the overall classification performance, particularly the accuracy of CogFuse-MobileViT across all categories, is remarkably high with minimal misjudgments, demonstrating that the model has enhanced discrimination ability for disease grades and performs optimally in distinguishing the four disease levels.

The Edge-Enhanced Feature Module (ECFM) effectively addressed Level 0 and Level 1 misclassification by capturing incipient lesion features. Baseline analysis revealed substantial false negatives for early-stage lesions, with Level 1 and Level 0 misclassification reaching 32%. ECFM reduced this rate to 8%, demonstrating its capacity to resolve texture confusion through enhanced edge contour delineation. Similarly, the Adaptive Multi-Scale Dilated Convolution Module (AMSDDICM) significantly mitigated Level 2 and Level 3 mutual misclassification. AMSDDICM drastically reduced both errors by enhancing local fine-grained features in mid-stage lesions (Level 2) while strengthening global fusion features in late-stage coalesced lesions (Level 3). This resolves grading ambiguity arising from the morphological continuum of lesion progression.

**Figure 13:**
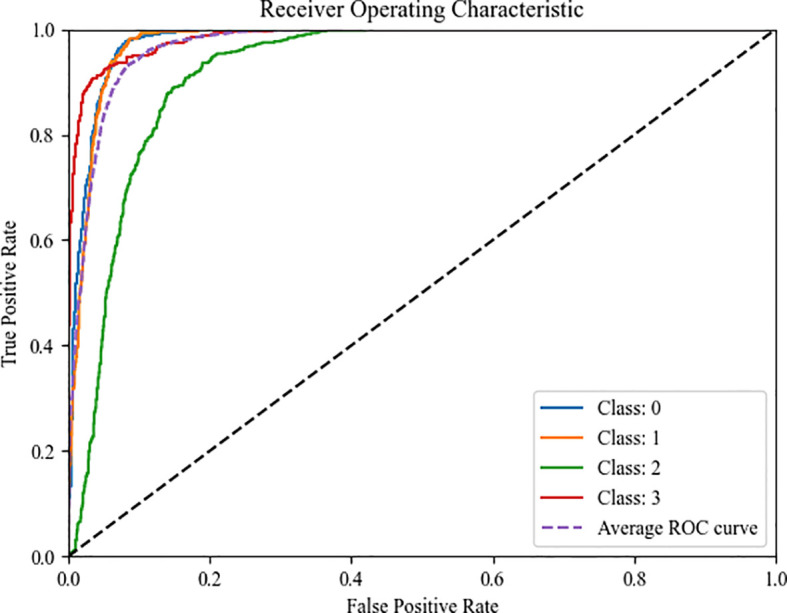
ROC curves of the four different severity levels for CogFuse-MobileViT.

To establish a comprehensive performance evaluation framework and quantify the model’s overall discriminative capability, **Figure 13** presents the Receiver Operating Characteristic (ROC) curves of CogFuse-MobileViT across four severity levels. These curves compare the True Positive Rate (TPR) against the False Positive Rate (FPR) by dynamically adjusting the classification threshold. The Area Under the Curve (AUC) serves as the primary performance metric, where a higher value indicates stronger classification ability. Notably, the AUC values for all categories significantly exceed the level of random guessing (AUC=0.5), fully demonstrating that the model possesses robust discriminative capability across different severity levels.

### 3.5 TSNE visualization of features extracted by different models

**Figure 14:**
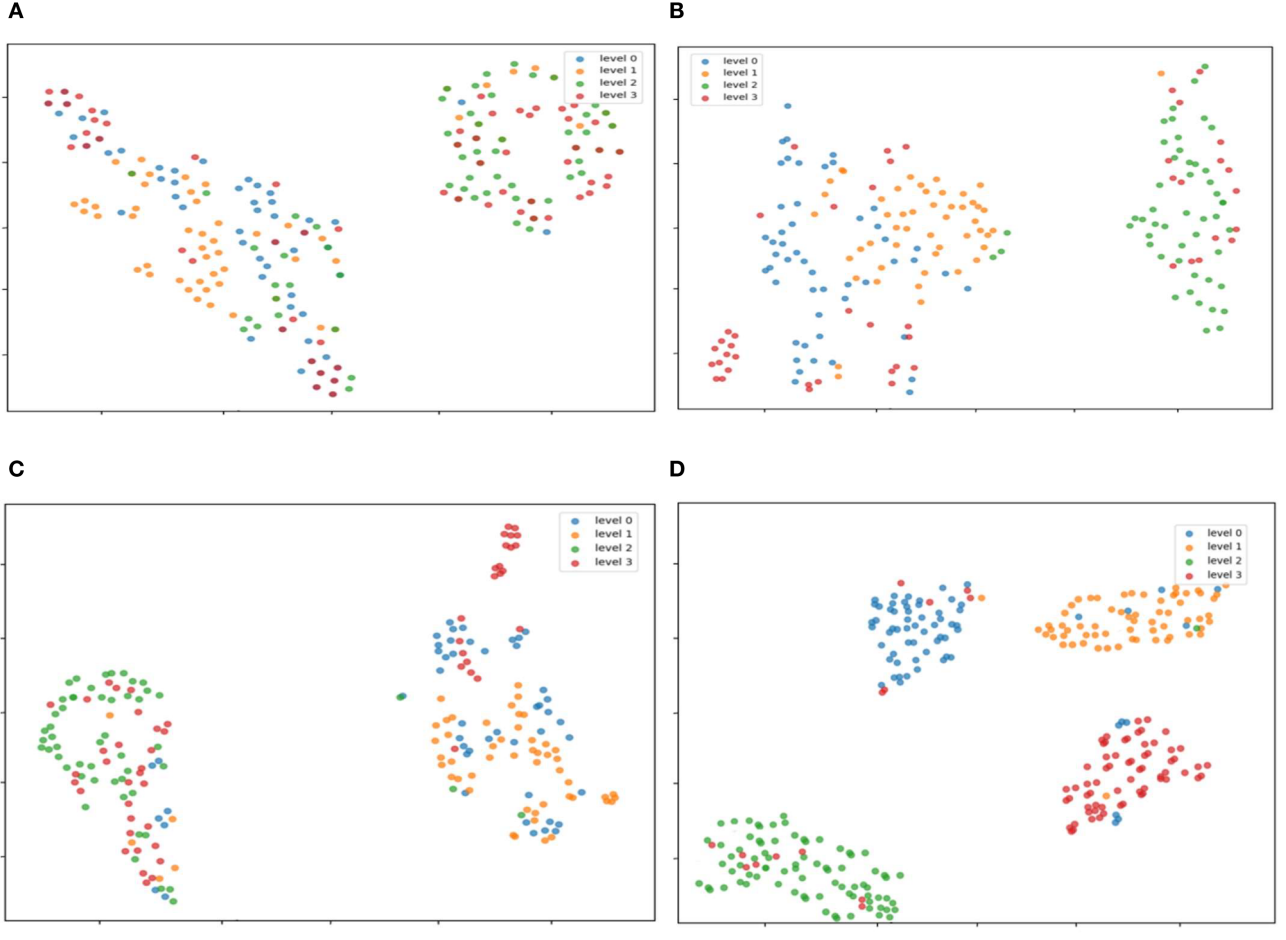
TSNE visualization of features extracted by different models. After the three modules work synergistically, the boundaries of feature clustering between Level 0 (healthy) and Level 1 (early-stage) are significantly clearer, which verifies the effectiveness of edge-texture collaborative learning **(A)** The original model was trained for 20 epochs; **(B)** The original model was trained for 50 epochs; **(C)** The original model was trained for 100 epochs; **(D)** The improved model was trained for 100 epochs.

The TSNE visualization of features extracted from the models is shown in **Figures 14A–D**. This study analyzed the features of the original model at the 20th, 50th, and 100th training epochs, as well as the improved model at the 100th epoch. After 20 epochs of training, the original model showed a highly discrete feature distribution. Limited by the number of training epochs, the model failed to fully learn discriminative features, resulting in insufficient distinction between categories and significant overlap among different classes. This indicates that under this training intensity, the original model’s ability to capture meaningful patterns was relatively limited. When the original model was trained to 50 epochs, compared with (a), the feature aggregation significantly improved. However, inter-class overlap still existed, suggesting that although prolonged training aided feature learning, the original model’s architecture had inherent defects in achieving clear feature separation. At 100 epochs of training, the original model exhibited more prominent feature clustering. Nevertheless, some regions still lacked clear separation between different categories, indicating that even after prolonged training, the original model faced challenges in maximizing inter-class distance and intra-class compactness. In Figure (d), the improved model after 100 epochs of training demonstrated a more superior feature distribution: each category was tightly clustered, achieving high intra-class compactness, while distinct boundaries between different categories were established, resulting in significant inter-class distance. This suggests that the model improvements effectively enhanced its ability to extract discriminative features, greatly reduced feature ambiguity, and improved feature discriminative power. Compared with the original model, it showed stronger feature discrimination and optimization potential.

### 3.6 Radar chart for comparison of classification performance between original and improved models

**Figure 15:**
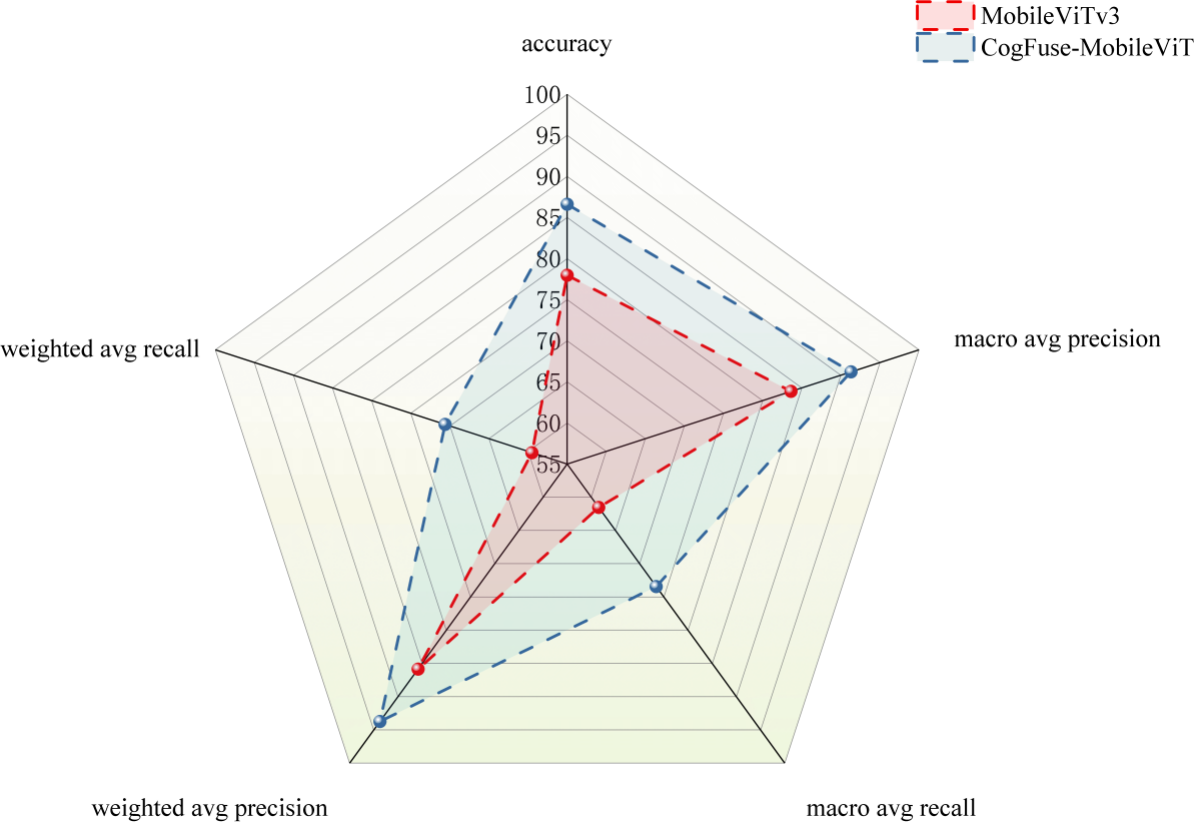
Multi-Metric Comparison Between MobileViTv3 and Improved CogFuse-MobileViT Model.

As shown in **Figure 15**, a radar chart compares the performance of the original model and the improved CogFuse-MobileViT model across multiple evaluation metrics. In terms of accuracy, CogFuse-MobileViT exhibits significantly higher values than the original MobileViTv3 model, indicating that the improved model achieves overall higher classification accuracy. Macro-average precision, which considers the average precision of each category, clearly shows that CogFuse-MobileViT also performs better, meaning it has superior precision across all categories. Meanwhile, CogFuse-MobileViT also demonstrates better macro-average recall. When examining weighted-average precision and weighted-average recall—metrics that account for class imbalance—CogFuse-MobileViT outperforms MobileViTv3 in both, indicating that in practical applications, even with uneven class distribution, the CogFuse-MobileViT model can deliver better performance. These results further demonstrate the model’s reliability and practicality.

### 3.7 Public data set experiment

To further validate the efficacy and generalizability of the proposed CogFuse-MobileViT model, experiments were conducted on the public dataset AppleLeaf9 (Yang et al., 2022). This dataset comprises healthy apple leaves and eight categories of apple leaf diseases captured in field environments without restrictions on imaging angles or noise interference. The dataset was partitioned into training and test sets at an 8:2 ratio. All images were resized to 224×224 pixels to optimize deep learning model training efficiency. Following the hyperparameter configurations specified in “Experimental Parameters and Evaluation Metrics”, both the baseline MobileViTv3 and CogFuse-MobileViT models were trained and evaluated.

Cross-species validation demonstrates that the CogFuse-MobileViT model delivers exceptional performance on the AppleLeaf9 dataset. As presented in **Table 9**, the model comprehensively surpasses the baseline MobileViTv3 across all four core metrics: precision increases by 0.16 percentage points, recall achieves a breakthrough improvement of 3.45 percentage points, F1-score rises by 1.18 percentage points, and accuracy elevates by 1.14 percentage points. The synergistic enhancement in both precision and recall signifies that performance gains stem from strengthened feature discriminability rather than metric trade-off compromises. The remarkable percentage points recall gain substantially mitigates leaf disease omission rates, while the holistic advance in F1-score further attests to the model’s robustness. These results collectively validate the generalizability of CogFuse-MobileViT’s core innovations in agricultural fine-grained disease grading, establishing a transferable paradigm for small-target pathology identification across plant species.

**Table 9:** Experimental results of the original model and CogFuse-MobileViT Model on Appleleaf9 data set.

Model	Precision (%)	Recall (%)	F1 (%)	Accuracy (%)
MobileViTv3	95.87	89.91	92.89	95.08
CogFuse-MobileViT	**96.03**	**92.36**	**94.07**	**96.22**

The bold font is used to emphasize the performance characteristics of the improved model on public datasets.

## 4 Conclusion

This study addresses the challenges in precise grading of walnut leaf brown spot disease. By adopting dynamic feature filtering, edge gradient reinforcement, and multi-scale morphological adaptation, it effectively resolves three key limitations of existing hybrid architectures and the baseline model MobileViTv3 in plant disease grading weak capability in capturing blurred edges, poor multi-scale adaptability, and interference from healthy tissues. Experimental results show that the model achieves an 86.61% grading accuracy on a dataset encompassing diverse lighting conditions, cultivars, and lesion stages, representing a 7.80% improvement over the baseline MobileViTv3 and outperforming nine state-of-the-art models. At the same time, this study confirmed that the performance improvement of CogFuse-MobileViT was statistically significant and stable through strict statistical tests, providing a reliable method for accurate classification of walnut leaf spot disease. The constructed image dataset and proposed grading re-measurement method lay the foundation for accuracy. This approach provides a new paradigm for small-target disease grading, with core modules transferable to multi-crop disease recognition. Beyond disease classification, context-aware AI frameworks demonstrate significant efficacy in agricultural management (Acharya et al., 2022). IoT systems dynamically adjust fertilization recommendations by integrating real-time soil-crop data, while salinity-corrected evapotranspiration (ETs) models optimize irrigation strategies for saline-alkali soils (Li et al., 2023; Su et al., 2023). Similarly, multimodal fusion techniques enhance reference evapotranspiration (ETo) prediction accuracy, facilitating precision water allocation (Jo et al., 2021; Rustia et al., 2023). These breakthroughs collectively validate the robust capability of adaptive feature processing in complex agricultural environments—a core principle that resonates with our dynamic weighting strategy for disease feature extraction. Future research should therefore integrate cross-modal and cross-domain capabilities to establish multidimensional assessment systems and regional dynamic monitoring frameworks, thereby delivering comprehensive technical solutions for small-target disease control.

## 5 Discussion and future work

Compared with existing methods, this study overcomes the limitation of traditional deep learning models relying on fixed convolution kernels, enabling adaptive capture of lesion features with multi-shaped convolution kernels to accurately extract edge textures of circular micro-lesions and regional contours of irregular lesions. Although the constructed multi-source dataset covers diverse lighting conditions, cultivars, and lesion development stages, the homogeneous internal features of severe diseases lead to limited recall rate. Meanwhile, when lesions are severely overlapped or mixed with mechanical damage, insect damage, or other types of injuries, the discriminative accuracy of the model is affected. For ultra-small lesions, the local feature information is too weak and easily overlooked, and the computational efficiency still needs optimization on some hardware platforms. Furthermore, the adaptability of the current model in extreme field scenarios, such as high-density occlusion and compound damage from pests and diseases, remains to be further verified. In these scenarios, the coupling of complex interference factors exacerbates feature confusion, affecting grading reliability.

To address these issues, future work will focus on the following research directions. Future research will focus on enhancing the model’s performance in complex field environments through multiple synergistic strategies. This includes constructing multi-interference factor coupled datasets that incorporating insect holes, lesions, and soil adhesion to strengthen robustness against extreme field disturbances; introducing attention-based multi-damage feature decoupling modules and dedicated micro-lesion enhancement modules combining super-resolution and feature interpolation to improve discriminability in complex scenarios and for tiny lesions; optimizing dynamic weight calculations via approximate computation or hardware-friendly reconstruction, while developing lightweight real-time deployment frameworks integrated with UAV near-ground sensing technology to boost efficiency and practical monitoring coverage; and establishing cross-crop pathological transfer learning mechanisms, extending the model to multi-crop disease recognition tasks with self-supervised pre-training to enhance algorithm universality and low-contrast lesion feature mining capabilities. Through the above research, it is expected to further improve the applicability and practicality of the model in complex field environments, promoting the development of intelligent plant disease grading technology towards more accurate, efficient, and universal directions.

## Data Availability

The raw data supporting the conclusions of this article will be made available by the authors, without undue reservation.
